
JOURNAL INFORMATION
==============================
NLM Title Abbreviation: ScientificWorldJournal
Journal ID: ScientificWorldJournal
NLM Journal ID: 101131163
Journal ID: TSWJ

The Scientific World Journal

ISSN: 2356-6140
EISSN: 1537-744X
Publisher: Wiley

ARTICLE INFORMATION
==============================
PMCID: PMC6084525
PMID: 30147592
DOI: 10.1100/tsw.2001.122
Article ID: 843017
Article version: 1
Subjects: Short Report

Pyrrolo-1,5-Benzoxazepines Induce Apoptosis in Chronic Myeloid Leukemia (CML) Cells by Bypassing the Apoptotic Suppressor BCR-ABL

Mc Gee Margaret M. 1 2 *
Campiani Giuseppe 3
Ramunno Anna 4
Fattorusso Caterina 4
Nacci Vito 3
Lawler Mark 5
Williams D. Clive 2
Zisterer Daniela M. 2
1 Biochemistry of Department Trinity College, Dublin 2, Ireland
2 Department of Biochemistry Trinity College, Dublin, Ireland
5 Department of Haematology Sir Patrick Dun Research Labs. St. James's Hospital and Trinity College, Dublin, Ireland
3 Dipartimento Farmaco Chimico Tecnologico Universita' degli Studi di Siena, Siena, Italy
4 Dipartimento di Scienze Farmaceutiche Universita' degli Studi di Salerno, Fisciano, Italy
*Margaret M. Mc Gee: mmcgee@tcd.ie
Publication date: 2001
Electronic publication date: 2001 Dec 19
Volume: 1
First page: 109
Last page: 109
Copyright: Copyright © 2001 Margaret M. Mc Gee et al.
Copyright year: 2001
License: This is an open access article distributed under the Creative Commons Attribution License, which permits unrestricted use, distribution, and reproduction in any medium, provided the original work is properly cited.
License URL: https://creativecommons.org/licenses/by/3.0/

Funding: National Pharmaceutical Biotechnology Centre

==============================
Miami *Nature Biotechnology* Short Reports
*TheScientificWorld* (2001) 1(S3), 109SR
ISSN 1532-2246; DOI 10.1100/TSW.2001.122

### PYRROLO-1,5-BENZOXAZEPINES INDUCE APOPTOSIS IN CHRONIC MYELOID LEUKEMIA (CML) CELLS BY BYPASSING THE APOPTOTIC SUPPRESSOR BCR-ABL

Margaret M. Mc Gee*, Giuseppe Campiani, Anna Ramunno, Caterina Fattorusso, Vito Nacci, Mark Lawler, D. Clive Williams, Daniela M. Zisterer

Department of Biochemistry, Trinity College, Dublin 2, Ireland
* mmcgee@tcd.ie

**INTRODUCTION.** Chronic myeloid leukemia (CML), which accounts for 20% of all leukemias, expresses the transforming oncogene, *bcr-abl*. Expression of *bcr-abl* results in the production of an abnormal tyrosine kinase and is reported to confer resistance against apoptosis induced by many chemotherapeutic agents. Recently a novel series of pyrrolo-1,5-benzoxazepines (PBOXs) were synthesised (Campiani *et al*., 1996) and some of these compounds induce apoptosis in a number of cancerous cells (Zisterer *et al*., 2000). In this study, a number of these novel pyrrolo-1, 5-benzoxazepines were found to induce apoptosis in CML cells. We examined whether Bcr-Abl becomes downregulated and whether its protein tyrosine kinase activity is altered during apoptosis (Mc Gee *et al.,* in press).

**METHOD.** Cells were cytocentrifuged onto slides and stained with eosin Y and methyl blue. Apoptotic cells were characterised by cell shrinkage, membrane blebbing, nuclear condensation and DNA fragmentation (Fig. 1). Levels of Bcr-Abl expression, protein tyrosine phosphorylation and PARP cleavage were measured by Western blot. Caspase 3-like protease activity was measured using a substrate, Ac-DEVD-AMC, which is cleaved and fluorogenic AMC released.

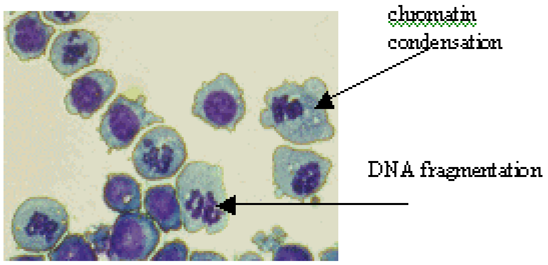

**RESULTS**. A representative pyrrolo benxozaxepine, PBOX-6, was found to induce 40-50% apoptosis in CML cells in a time and dose dependent manner (Fig. 2). Downregulation of Bcr-abl was not detected and the tyrosine phosphorylation status of proteins was unchanged up to 24 hours following treatment with PBOX-6. Caspase 3-like proteases were activated in K562

and LAMA 84 cells, but not in KYO.1 cells although apoptosis was induced to the same extent. Pretreatment of cells with a caspase 3-like inhibitor, Z-DEVD-fmk, prior to PBOX-6 inhibited caspase 3-like protease activity, but failed to prevent against apoptosis.

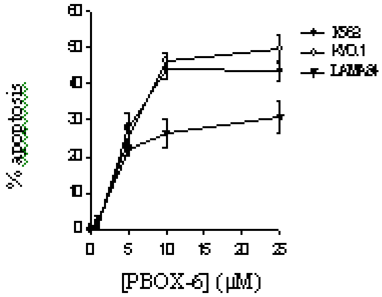

**DISCUSSION.** We have shown that PBOX-6 is a potent inducer of apoptosis in CML cells and is able to bypass Bcr-Abl mediated resistance. Downregulation of Bcr-Abl did not accompany but rather followed the induction of apoptosis. The tyrosine phosphorylation status of proteins remained unchanged up to 24 hours following treatment with PBOX-6. These results suggest that a reduction in Bcr-Abl expression, or inhibition of tyrosine kinase activity is not the only mechanism by which cells can escape the anti-apoptotic effect of the *bcr-abl* gene. Activation of caspase 3-like proteases is not required for the induction of apoptosis by PBOX-6 in the CML cells examined. Results from this study suggest the potential of this compound as a novel anti-cancer agent for the treatment of CML.

**ACKNOWLEDGEMENTS.** This study was supported by BioResearch Ireland, National Pharmaceutical Biotechnology Centre.

**REFERENCES.**

1. Ciampiani, G., Nacci, V., Fiorini, I., DeFilippis, M.P., Garofalo, A., Ciani, S.M., Greco, G., Novellion, E., Williams, D.C., Zisterer, D.M., Woods, M.J., Mihai, C., Manzoni, C., and Mennini, T. (1996) J. Med. Chem. 39, 3435-3450
2. Zisterer, D.M., Campiani, G., Nacci, V., and Williams, D.C. (2000) J. Pharmacol. Exp. Ther. 293, 48-59
3. Mc Gee, M.M., Campiani, G., Ramunno, A., Fattorusso, C., Nacci, V., Lawler, M., Williams, D.C., and Zisterer, D.M. J. Pharmacol. Exp. Ther. (in press)
